# The Role of Social Media Advertisement and Physical Activity on Eating Behaviors among the General Population in Saudi Arabia

**DOI:** 10.3390/nu16081215

**Published:** 2024-04-19

**Authors:** Sara Aleid, Najim Z. Alshahrani, Safa Alsedrah, Ana Branca Carvalho, Maria João Lima, Edite Teixeira-Lemos, António Raposo

**Affiliations:** 1 Preventive Medicine Division, Prince Sultan Military Medical City, Riyadh 11159, Saudi Arabia; sarah.aleid@hotmail.com (S.A.); salsedrah@psmmc.med.sa (S.A.); 2 Department of Family and Community Medicine, Faculty of Medicine, University of Jeddah, Jeddah 21589, Saudi Arabia; 3 ESTGL & CISeD—Research Centre in Digital Services, Polytechnic University of Viseu, 3504-510 Viseu, Portugal; acarvalho@estgl.ipv.pt; 4 CERNAS-IPV Research Centre, Polytechnic University of Viseu, 3504-510 Viseu, Portugal; mjoaolima@esav.ipv.pt (M.J.L.); etlemos3@gmail.com (E.T.-L.); 5 CBIOS (Research Center for Biosciences and Health Technologies), Universidade Lusófona de Humanidades e Tecnologias, Campo Grande 376, 1749-024 Lisboa, Portugal

**Keywords:** eating behaviors, food advertisement, physical activity, social media advertisement

## Abstract

Over the past few decades, people in Saudi Arabia have become less inclined to adopt active lifestyles and healthy eating habits due to the increasing use of digital technologies such as social media. The objective of this online-based cross-sectional study was to assess the role of social media food advertisements and physical activity on eating behaviors among the general population in Saudi Arabia (n = 471). Data were collected through a structured questionnaire, which consisted of four parts: (i) sociodemographic information, (ii) attitude towards social media, (iii) eating behaviors-related information, and (iv) exposure to and engagement with social media advertisements. The study’s outcome variable, eating behaviors (healthy vs. unhealthy), was assessed using the following question: “Are you on a healthy diet (such as a balanced diet, keto, or low carb)?” A multiple binary logistic regression analysis was performed to investigate the factors that influence unhealthy eating behaviors. Approximately 79.6% of the participants had unhealthy eating behaviors. Participants who were not involved in daily physical activity were more likely to have unhealthy eating behaviors compared to their counterparts (adjusted odds ratio, AOR = 4.86). Participants who watched food ads on social media channels 1–3 times a week (AOR = 2.58) or daily (AOR = 3.49) were more likely to have unhealthy eating behaviors than their counterparts. Participants whose appetite to try foods increases always (AOR = 1.42) or usually (AOR = 2.88) after viewing ads on social media were more likely to have unhealthy eating behaviors. These findings suggest that policymakers should take immediate action to regulate food advertising policy to promote a healthy food environment across the country. Saudis should be encouraged to engage in more physical activity, which could support the maintenance of healthy eating patterns and lifestyles.

## 1. Introduction

Over the past three to four decades, the world has significantly shifted into the stage of a nutrition transition, which is characterized by the high consumption of ultra-processed foods and notable declines in physical activity [1]. This stage is accompanied by a rapid rise in the prevalence of overweight, obesity, and other nutrition-related non-communicable diseases such as diabetes, hypertension, etc. [2]. There are no exceptions for Eastern Mediterranean countries and Gulf territories (such as Saudi Arabia, Iran, Kuwait, the United Arab Emirates, Qatar, and others); these regions are experiencing the nutrition transition [3,4,5,6,7,8,9,10]. For example, the United Arab Emirates has witnessed a rapid economic growth and nutritional shift that has had a substantial impact on the population’s lifestyle and eating habits, as evidenced by rising rates of obesity, diabetes, and metabolic syndrome [6,10,11]. In addition, Kuwaitis’ nutrition transition is characterized by increasing rates of overweight and obesity, excessive caloric food consumption, and the low intake of micronutrients and fiber [5]. Similarly, the Kingdom of Saudi Arabia is going through this transition, with a drop in the consumption of fresh produce (fruits and vegetables) and an increase in the consumption of processed foods and animal products [12]. As a consequence, the burdens of overweight and obesity are rising among adults, adolescents, and children in Saudi Arabia [3,13].

Eating behaviors can be influenced by many external factors such as food advertisements via television [14] and social media [15]. Food advertising has been shown to have a significant impact on consumers’ dietary decisions, eating habits, and consumption patterns [16]. In the modern era, social media sites like Facebook, Twitter, etc., are used as food advertising platforms to increase brand and information reach [15,17]. However, high-calorie and nutrient-poor foods are mostly advertised on social media, which have detrimental health consequences [18]. In particular, younger adults are more likely than older adults to use social media to manage their diet and purchase food without checking food/nutrition labels.

In the Kingdom of Saudi Arabia, the use of social media is increasing rapidly, which accelerates the risk of social media addiction [19,20]. The remarkable increase in social media users from 7.6 million in 2014 to 29.5 million in 2022 indicates that social media platforms are proliferating across the country [19]. Approximately 98.43% of people aged 15–35 years had at least one account on social networking sites [21]. On average, Saudi Arabians spend more than three hours daily on social media [21]. Thus, it becomes a means of communication, a business platform, as well as a platform for digital food marketing. A significant shift in food marketing/advertising has been brought about by the introduction of smartphones and tablets, as well as increased internet activity in the country. In most cases, social media advertisements frequently persuade consumers to buy or consume unhealthy foods and beverages. For example, a recent study conducted among university students in Saudi Arabia showed that participants who purchased foods/drinks more frequently after viewing such ads consumed higher amounts of potato chips and fast foods [22]. According to a study by Aldossari and Al-Mahish (2021) that focused on social media preferences for determining Saudi Arabian residents’ eating habits, the majority of respondents preferred unhealthy and nutrient-poor food products over healthy and nutrient-rich food [23]. In addition, there is evidence of the impact of social media influencers on food consumption among the Saudi population [24]. There is a knowledge gap in how food advertisements on social media affect eating behaviors among the general population in Saudi Arabia.

In addition, it is worth noting that a healthy diet combined with physical exercise prevents disease and maintains overall good physical and mental health and well-being. A systematic review reported that the majority of Saudi children, youth, and adults were not physically active enough to meet the standards for moderate-to-vigorous physical exercise [25]. Unhealthy dietary habits and suboptimal physical exercise can lead to various health complications, including obesity, cardiovascular diseases, diabetes, cancer, metabolic disorders, etc. [1].

To combat obesity and other non-communicable diseases, the Saudi government has developed and implemented different nutrition policies and guidelines over time [26]. Recently, the Saudi Food and Drug Authority (SFDA) has launched its healthy food strategy (HFS) as part of Saudi Vision 2030, which aims to improve healthy lifestyles and reduce the consumption of salt, sugar, saturated fatty acids, and trans fats [26]. The HFS is being implemented through a variety of nutritional reforms (such as displaying nutrition information in food and beverage businesses and allergens on food menus, etc.) as well as education campaigns [26]. As Saudi Arabia is committed to “Health in All Policies” and “Vision 2030” (including the transformation and strengthening of the healthcare system), understanding the determinants of eating behavior will aid the country’s disease prevention strategies.

Since eating behaviors and food choices are complex and multifactorial, the most important step before proposing interventions is to determine the factors that influence individual food preferences or behaviors [27]. Food marketing strategies and platforms can influence individual food choices, as evidenced by the literature [16]. Given the above-mentioned fact, it is of utmost importance to explore the effects of social media advertisements and lifestyle factors such as physical activity on eating behaviors among the population in Saudi Arabia. Therefore, the primary objective of this study was to assess the association between social media food advertisements and eating behaviors among the general population in Saudi Arabia. The secondary objective of this study was to identify socio-demographic and physical activity-related factors of eating behaviors. The findings of our study will raise public awareness about the behavioral effects of social media food advertisements, as well as inform and guide health experts and policymakers to take the necessary initiatives to promote healthy eating behaviors.

Initially, there were two hypotheses for this study: (i) different characteristics of food advertisements on social media, such as higher frequency of viewing food advertisements and increased appetite after watching food advertisements, would influence unhealthy eating behaviors; and (ii) reduced physical activity would be associated with unhealthy eating behaviors.

## 2. Materials and Methods

### 2.1. Study Design

An online-based cross-sectional survey was undertaken among the general population in Saudi Arabia. The participants were considered eligible as study participants when they met the following criteria: (i) being social media users and (ii) being adults (aged 18 years or older). Individuals with serious medical disorders mandating specific diets, such as celiac disease, diagnosed food allergies, and metabolic disorders, were not permitted to participate in this study.

### 2.2. Sample Size, Sampling and Data Collection Procedures

Using Cochran’s formula, the sample size was calculated. With the use of this formula, we can determine the optimum sample size (n) based on the desired accuracy (e), desired level of confidence (Z), and estimated proportion of the attribute present in the population (*p*). The sample size for this study was determined by taking into account the following hypotheses: (i) a 50% expected prevalence (*p* = 0.5), (ii) a 95% level of confidence (Z = 1.96), and (iii) a 5% margin of error (e = 0.05). The method of calculation is as follows:

$$\mathrm{Sample}\;\mathrm{size},\;n=\frac{z^{2}\times p\times (1-p)}{e^{2}}=\frac{1.96^{2}\times 0.5\times (1-0.5)}{0.05^{2}}=384.16\approx 385.$$

A minimum required sample of 385 participants was calculated for this study. We collected more samples than the calculated requirement to increase the generalizability of the study. Thus, the final sample size was 471.

Data were collected from online platforms such as social networking sites via a Google survey link containing the survey questionnaire. The survey link was distributed among participants from May to June 2023. A convenience sampling strategy was used to enroll the participants. Four trained data collectors, who were university students, were involved in the data collection. They were instructed on the survey contents, eligibility criteria, data collection approaches, and sampling techniques by the study team. They invited potential participants to participate in this study through the country’s most popular social media (such as Instagram, Snapchat, Twitter, etc.). Data collectors regularly monitored the survey process to achieve the minimal sample size or a greater sample size. They routinely counted the number of responses recorded and re-invited those who did not respond (7 days after the first invitation). The invitation letter for the survey began with a detailed explanation of the study’s objectives, and inclusion and exclusion criteria. Moreover, the participation was voluntary, and informed consent (electronic consent) was obtained from all the participants.

### 2.3. Survey Tool

A pre-tested structured questionnaire (Arabic version) was used to gather the data. The questionnaire was pre-tested among 20 participants to check the clarity of the questions. The pre-tested results were not included in the final analysis. Moreover, the content of the questionnaire was evaluated by three experts in the field of preventive medicine or family medicine. For each question, the expert had to determine whether the content measured by the question was “essential”, “useful but not essential”, or “not necessary” for measuring the construct. The content validity of the questionnaire was determined by a satisfactory agreement among the experts (i.e., when they considered the item essential). The questionnaire was finalized after appropriate modifications based on pre-testing and expert evaluations (for example, rewording some items to increase clarity).

The questionnaire was divided into four sections. In the first section, participants’ socio-demographic features such as age, gender, monthly income, marital status, place of living, level of education, job, and physical activity were included. The second section was about participants’ attitudes towards social media channels (such as type and average daily uses of social media). Considering the popularity of social media platforms and common sources of food advertising in Saudi Arabia, we focused on five specific social media platforms: Instagram, Snapchat, Twitter, TikTok and Youtube. The next section included eating behavior-related characteristics. Eating behaviors (healthy vs. unhealthy), the outcome variable of this study, were assessed by a question: “Are you on a healthy diet (such as a balanced diet, keto or low carb)?” Moreover, information on food sources, how many meals are consumed outside of the home, and how many meals are ordered via delivery applications was captured. Finally, seven questions based on the literature were used to assess the exposure to and engagement with social media advertisements [28,29]. This section includes information on the frequency of viewing food advertisements on social media, ways of interacting with food advertisements on social media (such as likes or shares), effects of advertisements on appetite, reliance on social media advertisements for daily food choices, etc.

### 2.4. Statistical Analysis

All statistical analyses were performed by Statistical Package for Social Science (SPSS, windows version 23) software. Descriptive (i.e., frequency and percentage) and analytical statistics were computed. Participants’ ages were categorized as ≤30 years, 31 to 35 years, and >35 years based on median age and cumulative percentage (age was collected via continuous measures during the survey). A chi-square or Fisher’s exact test was used to observe the distribution of eating behaviors across all predictor variables. A binary logistic regression model was fitted to determine the factors of unhealthy eating behaviors. Variables were added to the logistic regression model when the bivariate analysis showed that the *p* values were less than 0.20. All assumptions were checked regarding binary regression analysis. The fitness of the adjusted model was checked by Hosmer and Lemeshow test “chi-square (df) = 9.988 (8), *p*-value = 0.266”. Data were presented as odds ratio and 95% confidence interval. Finally, a *p* value of less than 0.05 was considered statistically significant.

### 2.5. Ethics

The study protocol was reviewed and approved by the Research Ethical Committee (IEC) of Prince Sultan Military Medical city (reference number: E-2091). Informed consent was obtained from all participants. The anonymity and confidentiality of their information were assured.

## 3. Results

### 3.1. Sociodemographic Characteristics

Participants’ sociodemographic characteristics are demonstrated in Table 1. A total of 471 participants constituted the sample of this study. Nearly half of the participants (48.6%) were aged between 20 to 30 years (median age = 31 years, interquartile range = 9). More than two-thirds (70.5%) of the participants were female, and the rest were male (29.5%). Approximately half of the participants (49.9) were married. Most of the participants (90.0%) lived in city areas. More than half of the participants had a bachelor’s degree (58.4%). Around one-third of the participants (28.9%) were involved in no physical activity and only twelve percent engaged in high-intensity activity.

### 3.2. Eating Behaviors

**Figure 1:**
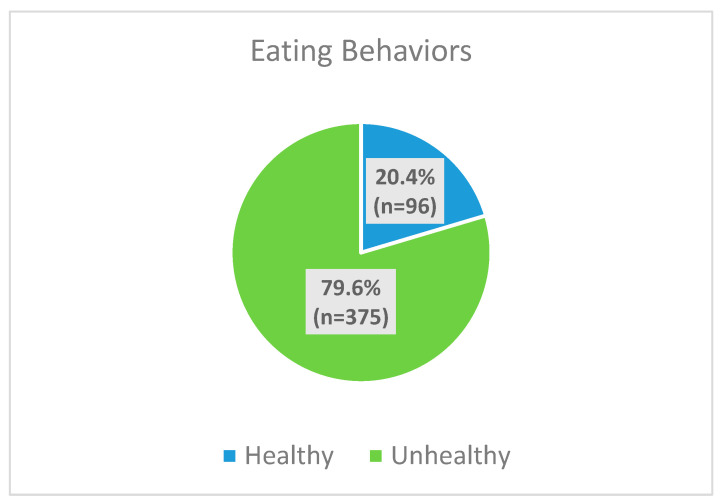
Prevalence of eating behaviors among study participants (n = 471).

Approximately 20.4% of the participants (n = 96) had healthy eating behaviors and 79.6% (n = 375) had unhealthy eating behaviors (Figure 1).

Three-quarters of the participants (74.4%) usually relied on delivery applications (36.9%) for their diet or ate in restaurants (37.4%). Eating behaviors-related information of study participants is shown in Table 2.

### 3.3. Food Advertisements on Social Media Platforms

Participants used or had an account on different social media apps. For example, 88.7% of the participants used Instagram and 87.5% of them had an account on Snapchat. The average daily use of social media among study participants is summarized in Table 3.

Nearly half of the participants (46.9%) reported that they daily or almost daily saw food ads on social media channels. Forty percent of the participants reported that their desire to try the food mentioned or similar products increases sometimes after watching food advertisement on social media platforms. Ten percent of the respondents’ desire to try the food mentioned or similar products increases always after watching food advertisements on social media platforms (Table 4).

### 3.4. Socio-Demographic and Food Advertisement-Related Factors of Eating Behaviors

Bivariate analysis shows that eating behaviors were significantly associated with participants’ physical activity levels (*p* < 0.001; Table 1). Moreover, the bivariate analysis found a significant association between eating behaviors and social media food advertisements, such as watching food ads on social media (*p* = 0.009), interacting with food ads on social media by clicking the “Like” button (*p* = 0.001), and increasing appetite to try advertised foods (*p* = 0.017) (Table 3).

Adjusted regression analysis revealed that participants who were not involved in daily physical activity were more likely to have unhealthy eating behaviors compared to their counterparts (adjusted odds ratio, AOR = 4.86, 95% CI: 2.11–7.17). Participants who watched food ads on social media channels one to three times a week (AOR= 2.58, 95% CI: 2.19–4.73) or daily (AOR = 3.49, 95% CI: 2.17–6.52) were more likely to have unhealthy eating behaviors than their counterparts. Participants whose desire to try foods always increases (AOR = 1.42–5.07, 95% CI: 1.42–5.07) or usually increases (AOR = 2.88, 95% CI: 1.58–5.53) after viewing the ads on social media were more likely to have unhealthy eating behaviors compared to their counterparts (Table 5).

## 4. Discussion

The results of this study show that just 20.4% of the participants engaged in healthy eating behaviors, while the majority (79.6%) of them had unhealthy eating behaviors. These findings indicate that the majority of the Saudi people had a poor diet quality, which is consistent with previous studies [12,30]. Sabur et al. (2022) conducted a study among Saudi adults and reported that 43.2% of participants did not meet the Ministry of Health requirements for any food group, while just 1.53% of individuals consumed the recommended amounts of each dietary group [30]. Another survey from Saudi Arabia revealed that just a small percentage of the Saudi population (adults aged ≥ years) adhered to the dietary guidelines, with only 5.2%, 7.5%, 31.4%, and 44.7% of individuals consuming the recommended portions of fruits, vegetables, nuts, and fish, respectively [12]. According to the Global Burden of Diseases, Injury and Risk Factors (GBD) study, Saudis consume more processed meat, red meat, total fatty acids and sodium than recommended for optimal health [31]. Our findings suggest collaborative initiatives to enhance eating behaviors among the Saudi population. Since diet-related factors are one of the major risk factors for mortality and morbidity, it is necessary to implement healthy diet interventions to improve dietary habits to reduce existing and future disease burdens in Saudi Arabia.

Our study found that participants’ eating behaviors were significantly influenced by different aspects of food advertisements on social media. Our regression model indicated that participants who viewed food advertisements on social media daily or one to three times per week had a higher risk of unhealthy eating behaviors. Furthermore, participants whose appetite to try foods always or usually increases after viewing the ads on social media were more likely to engage in unhealthy eating behaviors. These findings are somewhat comparable with other studies [22,28,29,32,33,34]. A recent study conducted among Turkish university students found that watching food videos on social media increases the urge to eat [33]. They suggested that watching food videos on social media may trigger psychological and physiological responses that increase the desire and frequency of food consumption [33]. Watching food videos may also stimulate the sensory memory of taste and smell, which increases salivation and hunger [33]. The influence of social media food advertisements on eating behaviors can be explained in two ways: (i) neurological mechanisms and (ii) marketing strategies, such as media influencers. Viewing high-calorie foods often precedes pleasurable and satiating intake in the neurological mechanism of human brains; hence, people have developed a drive to devour these foods or visually attend to foods [35,36,37]. Typically, searching for and consuming high-caloric foods makes people feel happy by releasing dopamine, which causes a sense of pleasure and satisfaction [38,39]. Another reason could be related to the use of social media influencers to promote food products, which influences people’s purchasing decisions and eating habits [24,40]. Moreover, it is evident that exposure to digital marketing has an impact on young people’s attitudes and habits toward a range of unhealthy products [41]. Our finding may be explained by the fact that there is a relationship between social media exposure and food cravings in young adults [42].

Due to the growing trend of social media users in Saudi Arabia [19], marketers use these platforms to advertise their food products, especially targeting young adults. However, the majority of food products that are advertised on social media are harmful to health, and marketers frequently disseminate erroneous information regarding nutritional value in order to capture customers [43,44]. A study conducted in Saudi Arabia reported that university students who used social media platforms like Snapchat, TikTok, and Instagram tended towards a higher consumption of potato chips, fast foods, sweets, and sugary drinks [22]. They also reported that obese individuals were more likely to buy foods/drinks after viewing relevant social media advertisements than their non-obese counterparts [22]. Our findings imply that food advertisements via social media have a great adverse influence on eating behaviors among the general population in Saudi Arabia.

It is highly recommended that policymakers should take the initiatives to regulate food advertising policy to create a healthy food environment across the county. The SFDA should take urgent action so that digital food marketers can demonstrate a positive gesture by advertising healthy foods and giving appropriate nutritional information related to those food products. Policymakers should develop and implement an awareness program on the use of social media, especially targeting young adults so that they can use it properly. For example, social media can be used as a nutrition education platform for healthy diet management by health professionals or dietitians. Furthermore, other stakeholders like public health practitioners and healthcare officials should sensitize the public to the health risks of a poor diet and social media addiction. Policymakers can also encourage social media influencers to advocate healthy diets and help boost public awareness about healthy diets and lifestyles. Comprehensive interventions or campaigns (covering the fundamentals of healthy eating habits and nutrition, as well as guidance on social media use) aimed at vulnerable populations such as young adults, students, and others should be implemented throughout the country. Future follow-up or qualitative studies are recommended to explore the effects of social media food marketing on eating attitudes and cognition among the general population, as well as adolescents and youth groups.

Another finding of this study revealed that participants who did not engage in daily physical activity were more likely to have unhealthy eating behaviors compared to their counterparts. It is well-established that diet and physical activity together are better than diet or physical activity alone to improve health outcomes such as metabolic health problems [45]. The importance of regular physical activity to one’s health and well-being has been shown by the growing body of research [46]. Nationally representative data show a low level of physical activity among Saudi adults who fulfilled physical activity guidelines [47]. These results imply that behavioral and lifestyle interventions should be developed to boost physical activity levels among Saudi citizens. Further qualitative or longitudinal studies that include a wide range of factors such as socio-demographic, behavioral, technological, etc., are recommended to identify the reasons for low physical activity among Saudis.

The study has some limitations, which should be considered when interpreting the results. Firstly, the cross-sectional nature of the study design cannot determine a causal relationship because exposure and outcome are assessed concurrently for each subject. Future studies with a longitudinal or qualitative approach may be able to claim causal correlations more effectively. Secondly, the generalizability of study results is limited due to convenience sampling techniques. Because we selected study participants based on convenience sampling (conventional), they may not accurately represent the study population, and sample estimates may not reflect the true effect. To increase the generalizability of the research findings, researchers should use probability sampling or homogeneous convenience samples (as a better alternative to traditional or heterogeneous convenience sampling) in their future research efforts [48]. Thirdly, in this study, the study population may not be representative in terms of the proportional participation of males and females; males accounted for only 30% of the total participants. The female-dominated sample population of the study could either understate or overstate its findings. The gender distribution of the samples, however, was similar to that in a previous Saudi study [49]. Finally, self-reported biases and social desirability biases may be present in the sample.

## 5. Conclusions

In our study sample, the majority had unhealthy eating behaviors (approximately 80%). Different parameters of food advertisement on social media, such as higher frequency of viewing food advertisements and increased appetite after watching food advertisements, as well as physical inactivity, were found to be associated with unhealthy eating behaviors. It is highly suggested that policymakers should take immediate action to regulate food advertising policy to promote a healthy food environment across the county. In addition, Saudis should be encouraged to engage in more physical activity, which could support the maintenance of healthy eating patterns and lifestyles. Future longitudinal or qualitative studies, including large and diverse samples, are recommended to help us better understand how eating behaviors are influenced by digital food marketing. Our findings suggest that the Saudi government and other departments should develop priority-based comprehensive educational and health interventions, including regarding healthy diet, lifestyle, and social media use, to improve healthy eating habits.

## Figures and Tables

**Table 1:** Sociodemographic characteristics of study participants (n = 471).

Variables	Total Sample		Eating Behavior; n (%)		*p* Value
	Frequency	Percentage	Healthy	Unhealthy	
Age (years)					0.932
≤30	229	48.6%	46 (20.1)	183 (79.9)	
31 to 35	111	23.6%	24 (21.6)	87 (78.4)	
>35	131	27.8%	26 (19.8)	105 (80.2)	
Gender					0.181
Male	139	29.5%	23 (16.5)	116 (83.5)	
Female	332	70.5%	73 (22.0)	259 (78.0)	
Monthly income (SAR)					0.588
<5000	121	25.7%	21 (17.4)	100 (82.6)	
5000 to 15,000	166	35.2%	37 (22.3)	129 (77.7)	
>150,000	184	39.1%	38 (20.7)	146 (79.3)	
Marital status					0.981
Single	236	50.1%	48 (20.3)	188 (79.7)	
Married	235	49.9%	48 (20.4)	187 (79.6)	
Place of living					0.188
City	424	90.0%	91 (21.5)	333 (78.5)	
Governorate	31	6.6%	4 (12.9)	27 (87.1)	
Village	16	3.4%	1 (6.3)	15 993.8)	
Place of residence					0.336
Living alone	54	11.5%	14 (25.9)	40 (74.1)	
Living with family	398	84.5%	80 (20.1)	318 (79.9)	
Live in group housing	19	4.0%	2 (10.5)	17 (89.5)	
Education					0.369
Up to high school	68	14.4%	11 (16.2)	57 (83.8)	
Bachelor’s degree	275	58.4%	54 (19.6)	221 (80.4)	
Post graduate	128	27.2%	31 (24.2)	97 (75.8)	
Job status					0.364
Health care worker	178	37.8%	39 (21.9)	139 (78.1)	
Non-health care worker	142	30.1%	32 (22.5)	110 (77.5)	
No employed	151	32.1%	25 (16.6)	126 (83.4)	
Daily physical activity					**<0.001**
No physical activity	136	28.9%	13 (9.6)	123 (90.4)	
Moderate activity	277	58.8%	63 (22.7)	214 (77.3)	
High intensity activity	58	12.3%	20 (34.5)	38 (65.5)	

Bold values indicate statistical significance (*p* < 0.05).

**Table 2:** Eating behaviors-related characteristics of study participants (n = 471).

Variables	Total Sample		Eating Behaviors; n (%)		*p* Value
	Frequency	Percentage	Healthy	Unhealthy	
Usually, on which source do you rely in your diet?					0.089
Home cooked	36	7.6	7 (19.4)	29 (80.6)	
Delivery applications	174	36.9	46 (26.4)	128 (73.6)	
Eat in restaurants	176	37.4	28 (15.9)	148 (84.1)	
Meals subscriptions	85	18.1	15 (17.6)	70 (82.4)	
In the past month, how many meals did you eat outside of your home?					0.227
Nothing in the last month	38	8.1	7 (18.4)	31 (81.6)	
1–3 times in the last month	179	38.0	45 (25.1)	134 (74.9)	
1–3 times a week	171	36.3	28 (16.4)	143 (83.6)	
Daily or almost daily	83	17.6	16 (19.3)	67 (80.7)	
In the past month, how many meals did you order via delivery applications?					0.391
Nothing in the last month	96	20.4	23 (24.0)	73 (76.0)	
1–3 times in the last month	186	39.5	40 (21.5)	146 (78.5)	
1–3 times a week	130	27.6	20 (15.4)	110 (84.6)	
Daily or almost daily	59	12.5	13 (22.0)	46 (78.0)	

**Table 3:** Average daily use of social media among study participants.

Social Media	Response (%)		
	No Account	≤2 h	≥2 h
Instagram	53 (11.3%)	284 (60.3%)	134 (28.5%)
Snapchat	59 (12.5%)	230 (48.8%)	182 (38.6%)
Twitter	118 (25.1%)	26 (57.1%)	84 (17.8%)
TikTok	205 (43.5%)	116 (24.6%)	150 (31.8%)
YouTube	103 (21.9%)	246 (52.2%)	122 (25.9%)

**Table 4:** Distribution of participants’ eating behaviors and social media food advertisements.

Variables	Total; n (%)	Eating Behaviors; n (%)		*p* Value
		Healthy	Unhealthy	
In the past month, how many food ads did you see on social media channels?				**0.009**
Nothing in the last month	44 (9.3)	24 (54.5)	20 (45.5)	
1–3 times in the last month	87 (18.5)	23 (26.4)	64 (73.6)	
1–3 times a week	119 (25.3)	21 (17.7)	98 (82.3)	
Daily or almost daily	221 (46.9)	28 (12.7)	193 (87.3)	
In the past month, how many times have you interacted with food ads on social media channels by clicking the “Like” button?				**0.001**
Nothing in the last month	296 (62.8)	45 (15.2)	251 (84.8)	
1–3 times in the last month	112 (23.8)	37 (33.0)	75 (67.0)	
1–3 times a week	40 (8.5)	8 (20.0)	32 (80.0)	
Daily or almost daily	23 (4.9)	6 (26.1)	17 (73.9)	
In the past month, how many times have you interacted with food ads on social media channels by clicking the “Share” button?				0.379
Nothing in the last month	310 (65.8)	52 (16.8)	258 (83.2)	
1–3 times in the last month	110 (23.4)	32 (29.1)	78 (70.9)	
1–3 times a week	39 (8.3)	9 (23.1)	30 (76.9)	
Daily or almost daily	12 (2.5)	3 (25.0)	9 (75.0)	
After watching the food advertisement, your appetite increases to try the food mentioned or similar products.				**0.017**
Always	50 (10.6)	06 (12.0)	44 (88.0)	
Usually	104 (22.1)	19 (18.3)	85 (81.7)	
Sometimes	192 (40.8)	37 (19.3)	155 (80.7)	
Rarely	97 (20.6)	23 (23.7)	74 (76.3)	
Never	28 (5.9)	11 (39.2)	17 (60.8)	
After watching the food advertisement, you decide to try the food mentioned or similar products.				0.673
Always	21 (4.5)	4 (19.0)	17 (81.0)	
Usually	74 (15.7)	11 (14.9)	63 (85.1)	
Sometimes	192 (40.8)	44 (22.9)	148 (77.1)	
Rarely	139 (29.5)	27 (19.4)	112 (80.6)	
Never	45 (9.6)	10 (22.2)	35 (77.8)	
In general, you rely on food advertisements in choosing your daily meals.				0.638
Always	8 (1.7)	2 (25.0)	8 (75.6)	
Usually	36 (7.6)	4 (11.1)	32 (88.9)	
Sometimes	111 (23.6)	23 (20.7)	88 (79.3)	
Rarely	152 (32.3)	30 (19.7)	122 (80.3)	
Never	164 (34.8)	37 (22.6)	127 (77.4)	
What is the application whose food ads you most depend on in choosing your daily meals?				0.288
Instagram	157 (33.3)	40 (25.5)	117 (74.5)	
Snapchat	134 (28.5)	25 (18.7)	109 (81.3)	
Twitter	20 (4.2)	6 (30.0)	14 (70)	
TikTok	92 (19.5)	14 (15.2)	78 (84.8)	
YouTube	68 (14.4)	11 (16.2)	57 (83.8)	

Bold values indicate statistical significance (*p* < 0.05).

**Table 5:** Binary logistic regression presenting the factors associated with unhealthy eating behaviors among study participants.

Variables	Adjusted Regression Model ^†^		
	Odds Ratio	95% CI (Lower-Upper)	*p* Value
Gender			
Male	1.28	0.71–2.31	0.418
Female	Reference		
Place of living			
City	0.25	0.02–2.04	0.193
Governorate	0.48	0.04–5.21	0.547
Village	Reference		
Physical activity			
No physical activity	4.86	2.11–7.17	**0.000**
Moderate activity	1.75	0.90–3.42	0.098
High-intensity activity	Reference		
Usually, on which source do you rely in your diet?			
Home cooked	0.89	0.30–2.67	0.843
Delivery Applications	0.64	0.32–1.31	0.226
Eat in restaurants	1.34	0.64–2.79	0.432
Meals subscriptions	Reference		
Frequency of watching food ads on social media channels			
Nothing in the last month	Reference		
1–3 times in the last month	1.78	0.85–2.49	0.082
1–3 times a week	2.58	2.19–4.73	**0.006**
Daily or almost daily	3.49	2.17–6.52	**<0.001**
Interact with food ads on social media channels by clicking the “Like” button			
Nothing in the last month	Reference		
1–3 times in the last month	0.35	0.20–1.60	0.170
1–3 times a week	0.72	0.28–1.97	0.480
Daily or almost daily	0.52	0.18–1.52	0.233
After watching food advertisement, appetite increases to try the food mentioned or similar products.			
Always	2.46	1.42–5.07	**0.002**
Usually	2.88	1.58–5.53	**0.009**
Sometimes	1.69	0.92–4.88	0.092
Rarely	1.16	0.39–1.49	0.193
Never	Reference		

^†^ Hosmer and Lemeshow test: chi-square = 9.988 (8), *p* = 0.266. Bold values indicate statistical significance (*p* < 0.05).

## Data Availability

The original contributions presented in the study are included in the article, further inquiries can be directed to the corresponding authors.
